# Hydrogen Sulfide Alleviates Cadmium-Induced Cell Death through Restraining ROS Accumulation in Roots of *Brassica rapa* L. ssp. *pekinensis*

**DOI:** 10.1155/2015/804603

**Published:** 2015-05-11

**Authors:** Liping Zhang, Yanxi Pei, Hongjiao Wang, Zhuping Jin, Zhiqiang Liu, Zengjie Qiao, Huihui Fang, Yanjie Zhang

**Affiliations:** School of Life Science, Shanxi University, Taiyuan 030006, China

## Abstract

Hydrogen sulfide (H_2_S) is a cell signal molecule produced endogenously and involved in regulation of tolerance to biotic and abiotic stress in plants. In this work, we used molecular biology, physiology, and histochemical methods to investigate the effects of H_2_S on cadmium- (Cd-) induced cell death in Chinese cabbage roots. Cd stress stimulated a rapid increase of endogenous H_2_S in roots. Additionally, root length was closely related to the cell death rate. Pretreatment with sodium hydrosulfide (NaHS), a H_2_S donor, alleviated the growth inhibition caused by Cd in roots—this effect was more pronounced at 5 *μ*M NaHS. Cd-induced cell death in roots was significantly reduced by 5 *μ*M NaHS treatment. Under Cd stress, activities of the antioxidant enzymes were significantly enhanced in roots. NaHS + Cd treatment made their activities increase further compared with Cd exposure alone. Enhanced antioxidant enzyme activity led to a decline in reactive oxygen species accumulation and lipid peroxidation. In contrast, these effects were reversed by hydroxylamine, a H_2_S inhibitor. These results suggested that H_2_S alleviated the cell death caused by Cd via upregulation of antioxidant enzyme activities to remove excessive reactive oxygen species and reduce cell oxidative damage.

## 1. Introduction

Recently, hydrogen sulfide (H_2_S) has become appreciated as an endogenous signaling molecule, after nitric oxide and carbon monoxide [1]. In the 1980s, H_2_S release in plants was discovered [2]. Some genes encoding these enzymes, which are responsible for endogenous H_2_S generation, were recently cloned in higher plants. Two cysteine desulfhydrases with the ability to decompose cysteine to pyruvate, ammonia, and H_2_S have been identified: L-cysteine desulfhydrase (LCD) with L-cysteine as substrate and D-cysteine desulfhydrase (DCD) with D-cysteine as substrate [3, 4]. Since then, Álvarez et al. reported a novel L-cysteine desulfhydrase, named DES1, which is an* O*-acetylserine(thiol)lyase homolog [5]. Some enzymes with similar function are being discovered, but detailed information remains limited.

As a signal molecule, H_2_S plays a vital role in regulating the growth and development of plants; moreover the important effects of H_2_S in plants response to some stresses have been intensely discussed in recent years. Increasing amounts of evidence illustrate the physiological functions of H_2_S in the growth and development of plants, such as enhancing photosynthesis, regulating seed germination, stomatal movement, root formation, and flower senescence [6–10]. In addition, H_2_S, as a pivotal role in plant response to environmental stimuli, such as improving drought resistance, coping with heat stress, enhancing freezing tolerance, and involvement in plants response to heavy metal, osmotic, and salt stresses, has also been reported [11–15]. The protective roles of H_2_S alleviating stresses have focused on promoting antioxidant capacity to decrease reactive oxygen species (ROS) accumulation or interacting with other signaling molecules. However, it is just the beginning of studying H_2_S-mediated stress responses, and the potential molecular mechanisms remain ambiguous.

Cadmium (Cd) is a major environmental pollutant and can be easily transported from the roots to other parts of plant. Cd also displays deleterious effects on seed germination, growing development, and photosynthesis [16, 17]. Treatment with high Cd concentrations can trigger programmed cell death (PCD) or necrosis in tobacco and* Arabidopsis* cell cultures [18, 19]. These negative effects of Cd were found to be mediated by ROS accumulation.

ROS including hydrogen peroxide (H_2_O_2_), superoxide radical (O_2_^•−^), hydroxyl radical, and single oxygen are generated unavoidably in processes of glycolysis and photosynthesis, which are important to energy production and storage strategies for aerobic microbes and plants. In plants, ROS are continuously produced predominantly in chloroplasts, mitochondria, and peroxisomes [20]. Under normal growth conditions, ROS are produced and maintained at a low level in vivo by a complex antioxidant system. Low molecular-weight antioxidants (ascorbic acid, glutathione reduced, carotenoids, and tocopherols) and antioxidant enzymes such as superoxide dismutase (SOD), ascorbate peroxidase (APX), peroxidase (POD), and catalase (CAT) in plant can scavenge ROS [21]. Under stress conditions, ROS formation exceeds the capacity of antioxidant system scavenging and results in oxidative stress. The cellular Cd intoxication mechanism is known to disturb redox homeostasis by indirectly stimulating ROS production in the train of oxidative damage through oxidizing lipids, proteins, DNA, and carbohydrates. Malondialdehyde (MDA), the product of lipid peroxidation, can cause membrane components cross-linking and polymerization [22]. In this way, the structure of cell membranes may be destroyed.

Our research concerns Chinese cabbage (*Brassica rapa* L. ssp.* pekinensis*) which has a close genetic relationship with* A. thaliana* and a clear genetic background. Because of having greater biomass than* A. thaliana*, the root growth relating index of Chinese cabbage can be detected more easily. The aim of this work is to provide more evidence for the potential mechanisms of H_2_S mitigation of developmental inhibition caused by Cd stress. We investigated changes on root elongation, ROS content, lipid peroxidation level, electrolyte leakage percentage (ELP), cell death, and DNA damage. The results showed that H_2_S could decrease cell death rate to alleviate Cd-induced growth inhibition through regulating the ROS balance in Chinese cabbage roots.

## 2. Materials and Methods

### 2.1. Plant Material and H_2_S Treatment

A commercial variety of Chinese cabbage (Jinyu75) was used. Plants were grown in nutrient soil : vermiculite (1 : 1, v/v) in growth chambers at 23°C with a photoperiod of 16/8 h (light/dark). Light (160 *μ*Em^−2^ s^−1^) was supplied by cool white fluorescent tube. At 3 days after emergence, seedlings were irrigated by cadmium chloride (CdCl_2_) solutions of different concentrations (0, 5, 10, and 20 mM). Gene expressions were determined after Cd exposure for 24 h. Root length, H_2_S production, and physiological indexes were determined after 48 h of Cd exposure. Pretreatment with sodium hydrosulfide (NaHS), a H_2_S donor, was performed for 24 h before Cd treatment. Pretreatment of 1 mM hydroxylamine, an inhibitor of H_2_S generation, was performed for 4 h before Cd treatment.

### 2.2. Detection of Cell Death

Cell death was measured according to the method of Turner and Novacky [23] with some modifications. Roots (2 cm long) were incubated in 0.25% Evans blue solution for 15 min and washed with water for 10 min. The trapped Evans blue was released from the roots by homogenizing with 1.0 mL of 80% alcohol. The homogenate was incubated for 15 min in a water-bath at 50°C and centrifuged at 10,000 ×g for 10 min. The absorbance of supernatant was measured at 600 nm and calculated on the basis of fresh weight.

### 2.3. Detection of DNA Fragmentation

The fragmented DNA was extracted with a DNA purification kit (Beyotime, C0008) according to the manufacturer's protocols. The eluent containing DNA was subjected to electrophoresis on a 1.5% agarose gel. DNA bands were observed and analyzed electrophoretically by an ultraviolet gel documentation system (Bio-Rad, USA).

### 2.4. Determination of H_2_S Production Rate

Endogenous H_2_S was determined by the method of Sekiya et al. [2] with some modifications. Roots were homogenized in 1 mL of 50 mM phosphate buffer solution (PBS, pH 7.0) containing 0.1 M EDTA and 0.2 M ascorbic acid. The homogenate was mixed with 0.5 mL of 1 M Tris-HCl in an Erlenmeyer flask to release H_2_S, and H_2_S was absorbed in a test tube containing 1% zinc acetate located in the bottom of the Erlenmeyer flask. After 15 min of reaction, 0.15 mL of 3.5 mM H_2_SO_4_ solution, containing 5 mM dimethyl-*p*-phenylenediamine, was added to the test tube and then 0.15 mL of 50 mM FeCl_3_ was added. After 15 min, absorption at 667 nm was measured.

### 2.5. Analysis of Transcript Levels

The total RNA of roots was extracted using RNAiso plus (Takara, D325A). The cDNA was synthesized using a Reverse Transcription System Kit (Takara, D6110A). Real-time PCR was performed by a real-time PCR (Bio-Rad, California, USA) detection system. The list of primers was supplied in Table S1 (see Supplementary Material available online at http://dx.doi.org/10.1155/2015/804603). All reactions were repeated independently at least three times. Statistical analysis was performed using iQ5 software (Bio-Rad, California, USA).

### 2.6. Measurement of Intracellular H_2_O_2_, O_2_^•−^, and ROS Levels

H_2_O_2_ and O_2_^•−^ were measured according to a previously described method [24] with some modifications. For cytochemical visualization of H_2_O_2_, intact roots were immersed in 1% solution of 3,3-diaminobenzidine, incubated at room temperature for 2 h, illuminated until appearance of brown precipitate, and then decolorized with 95% alcohol. For cytochemical visualization of O_2_^•−^, intact roots were immersed in a 0.1% solution of nitroblue tetrazolium in 50 mM PBS (pH 7.4), at room temperature, and illuminated until the blue formazan precipitate appeared and then decolorized with 95% alcohol. ROS was determined by fluorimetric assay. Root tissues were incubated with 25 mM 2′,7′-dichlorofluorescein diacetate at 37°C for 30 min. Results were observed by fluorescence microscopy (488 nm) and fluorescence intensity was analyzed with LSM 5 software (ZEISS, Germany).

### 2.7. Measurement of MDA and Electrolyte Leakage Percentage (ELP)

MDA content was assayed as described by Heath and Packer [25] with some modifications. Root tissues were homogenized with 5% trichloroacetic acid (TCA). After centrifuging at 10,000 ×g for 5 min, supernatant was mixed with 5% TCA containing 0.68% thiobarbituric acid. The mixture was heated at 98°C for 30 min and centrifuged at 7500 ×g for 5 min. The absorbance of the supernatant was measured at 532,600 and 450 nm.

ELP was measured using an electrical conductivity meter according to Lutts et al. [26]. Roots were washed with distilled water and 0.2 g of samples were placed in tubes containing 10 mL of distilled water and then incubated at 25°C for 1 h. Then the electrical conductivity of the bathing solution (EC1) was read. Samples were then placed in a water-bath at 95°C for 30 min and the second reading (EC2) was determined after cooling to 25°C. ELP was calculated as ELP = (EC1/EC2) × 100%.

### 2.8. Enzyme Extraction and Activity Measurements

Activities were analyzed by the methods described by Li et al. [27] with some modifications. Roots were homogenized in 1.5 mL of 50 mM PBS buffer containing 1 mM EDTA and 1% polyvinylpyrrolidone. The homogenate was centrifuged at 10,000 ×g for 10 min at 4°C and the supernatant was used to examine the activity of antioxidant enzymes.

Total SOD activity was measured colorimetrically at 560 nm, based on the ability of O_2_^•−^ generated by the riboflavin system under illumination to reduce nitroblue tetrazolium. CAT activity was determined at 290 nm for 3 min, using 1 mL reaction mixture containing 50 mM PBS buffer, 2% H_2_O_2_, and 50 *μ*L of supernatant. One unit of CAT activity was defined as a decrease of absorbance of 0.01 min^−1^. A mixture containing 50 mM PBS buffer (pH 7.0), 3% guaiacol, 2% H_2_O_2_, and 50 *μ*L of enzyme extract was used to measure POD activity at 470 nm for 3 min. POD activity was expressed as increase of absorbance of 0.01 per min as one enzyme unit. APX activity was determined as the decrease in *A*_290_ for 3 min in 1 mL of reaction mixture containing 50 mM PBS buffer (pH 7.0), 15 mM ascorbate, 30 mM H_2_O_2_, and 50 *μ*L of enzyme extract. One unit of APX activity was defined as a decrease of absorbance of 0.01 per min.

### 2.9. Statistical Analysis

All experiments were performed in triplicate. Data were presented as mean ± SE. One-way analysis of variance was used for multiple comparisons using SPSS 17.0 software (IBM SPSS, Chicago, USA).

## 3. Results

### 3.1. Cell Death due to Cd Stress Arrests Root Growth

**Figure 1:**
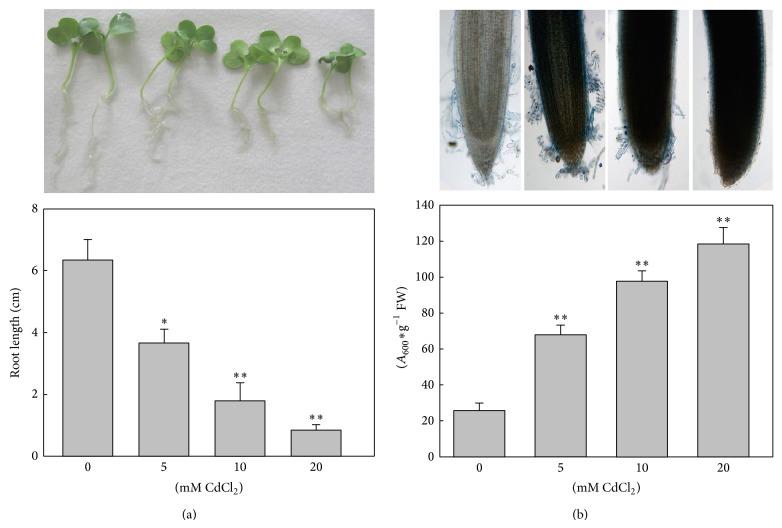
Influence of Cd stress on root development and cell death in Chinese cabbage roots. (a) Root growth under Cd stress. (b) Roots stained with Evans blue observed under a light microscope and quantitative analysis of cell death caused by Cd exposure. Three-day-old seedlings were exposed to different concentrations of Cd (0, 5, 10, and 20 mM) for 24 h. Data are mean ± SE of three independent repeats. Using one-way ANOVA and compared with controls, significance is shown by ^∗^ *P* < 0.05, ^∗∗^ *P* < 0.01.

To explore the negative effects of Cd exposure on Chinese cabbage roots, 3-day-old seedlings were treated with CdCl_2_ at increasing concentrations (0, 5, 10, and 20 mM) for 48 h. The root length was significantly (*P* < 0.05) inhibited by 5 mM CdCl_2_ treatment compared to controls (Figure 1(a)). Furthermore, growth inhibition of roots after Cd exposure decreased in a dose-dependent manner (*R* ^2^ = 0.9878, *P* < 0.05). With 10 and 20 mM CdCl_2_ treatment, the growth inhibitions were 71.56% and 86.52%, respectively. Moreover, the black spots and necrosis of the roots tips can be observed. Thus, 5 mM CdCl_2_ was chosen for further experiments.

Because Evans blue can stain the cell walls of dead cells, it was used to indicate dead cells. Roots treated with CdCl_2_ showed a deeper level of dye compared with controls, and the quantity of Evans blue extracted from roots sharply increased (Figure 1(b)). The content of Evans blue was 4.6 times higher for 20 mM CdCl_2_ treatment than controls. Furthermore, the length of roots was inversely related to the death rate of root cells (*y* = −16.681*x* + 130.24, *R* ^2^ = 0.9968, *P* < 0.05). These results demonstrated that Cd inhibited root growth due to causing the death of root cells.

### 3.2. H_2_S Is Involved in Response to Cd Stress

**Figure 2:**
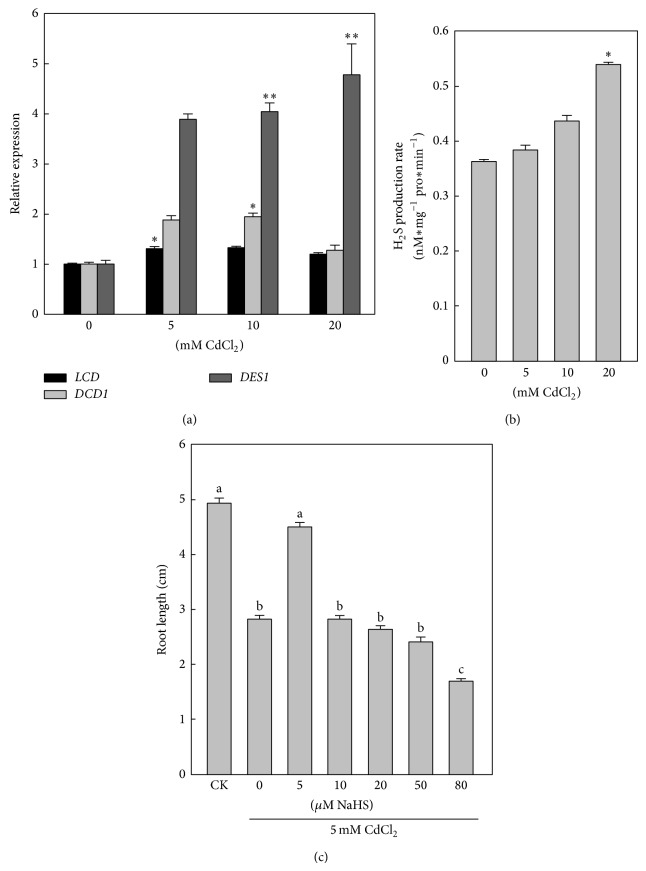
Emission of H_2_S and its positive effect on root growth inhibition caused by Cd stress in Chinese cabbage. (a) The expressions of H_2_S synthase-encoding genes* LCD*,* DCD1*, and* DES1* under Cd stress. (b) The production rate of H_2_S under increasing concentrations of Cd. (c) Effects of different concentrations of NaHS on root development under 5 mM Cd treatment. Three-day-old seedlings were exposed to different concentrations of Cd (0, 5, 10, and 20 mM) for 48 h. Data are mean ± SE of three independent repeats. Using one-way ANOVA and compared with controls, significance is shown by ^∗^ *P* < 0.05, ^∗∗^ *P* < 0.01; LSD was used for multiple comparisons; different letters indicate significant differences (*P* < 0.05).

To investigate the relationship between Cd stress and endogenous H_2_S in Chinese cabbage, the expression of genes* BraLCD*,* BraDCD1*, and* BraDES1* (Figure 2(a)) and the production rate of H_2_S were examined (Figure 2(b)). The endogenous H_2_S emission was stimulated by Cd stress. Firstly, after a range of concentrations (0, 5, 10, and 20 mM) of Cd treatment for 24 h, the relative expressions of* BraDCD1* and* BraDES1* were upregulated. However* BraLCD* expression level was not increased. The expression level of* BraDES1* differed significantly under the 5 mM treatment, and expression level under the 20 mM Cd treatment was 4.7 times of the control. Secondly, as Cd concentration increased, the H_2_S production also gradually increased.

H_2_S was applied to verify the role of H_2_S in enhancing plant tolerance to Cd stress. Seedlings were pretreated with different concentrations (5, 20, 50, 80, and 100 *μ*M) of NaHS and then exposed to Cd. Inhibition of root growth was markedly (*P* < 0.05) alleviated by 5 *μ*M NaHS, and the other concentrations of exogenous NaHS pretreatment had no significant effect and even showed some negative effects (Figure 2(c)). Thus, 5 *μ*M NaHS was used in further experiments.

### 3.3. H_2_S Influenced the Cd-Induced Cell Death and DNA Damage

**Figure 3:**
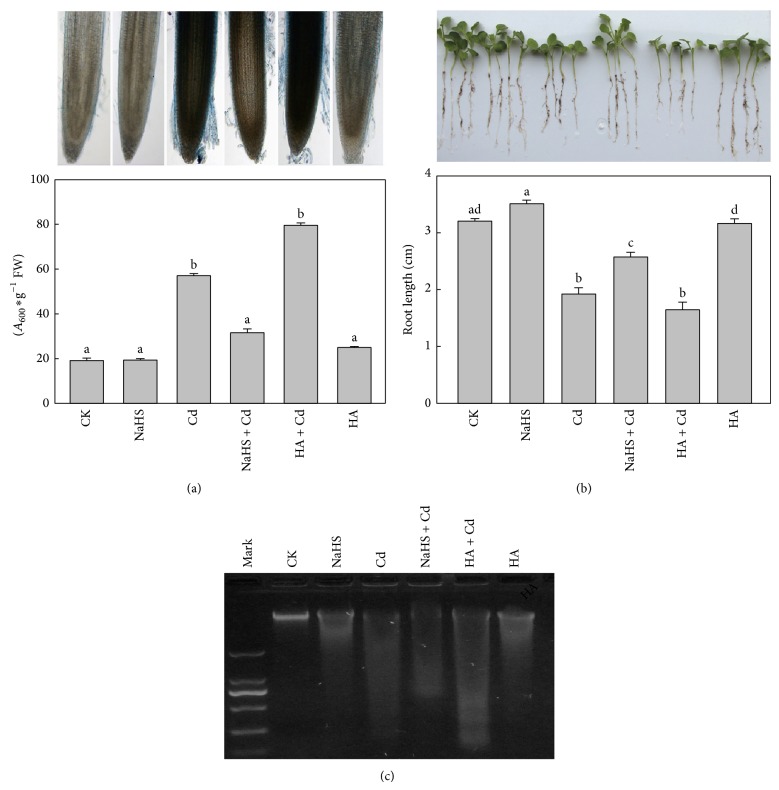
Effect of H_2_S on Cd-induced root cell death in Chinese cabbage. (a) Roots stained with Evans blue observed under a light microscope and quantitative analysis of cell death caused by Cd stress. (b) DNA damage analysis. (c) Root growth phenotypes. CK: control; NaHS: fumigated with 5 *μ*M NaHS for 24 h; Cd: 5 mM Cd treatment for 48 h; NaHS + Cd: seedlings fumigated with 5 *μ*M NaHS for 24 h and then treated with 5 mM Cd for 48 h; HA + Cd: seedlings treated with 1 mM HA for 4 h and then treated with 5 mM Cd for 48 h; HA: seedlings treated with 1 mM HA for 4 h. Data are mean ± SE of three independent repeats. LSD was used for multiple comparisons; different letters indicate significant differences (*P* < 0.05).

There were fewer dead cells in controls and the NaHS treatment alone, but Cd exposure notably increased cell mortality (Figure 3(a)). When seedlings were pretreated with 5 *μ*M NaHS for 24 h before 5 mM Cd treatment, the content of Evans blue was reduced by 31.9% (*P* < 0.05) compared with Cd treatment alone. The content of Evans blue differed significantly between Cd and HA + Cd treatments.

To confirm the above results concerning cell death, DNA-ladder assays were performed based on DNA or chromatin fragmentation after cell death. Genomic DNA degradation was not apparent in controls (Figure 3(b)). Strong nuclear-DNA random cleaving appeared with Cd treatment and weakened with NaHS + Cd treatment but it was more serious in the HA + Cd treatment.

A further phenotype test was carried out using exogenous H_2_S or HA to treat seedlings (Figure 3(c)). As expected, the NaHS pretreatment greatly improved root growth under Cd stress. The HA + Cd treatment greatly inhibited the elongation of roots.

### 3.4. H_2_S Lowered the Cd-Induced Accumulation of ROS

**Figure 4:**
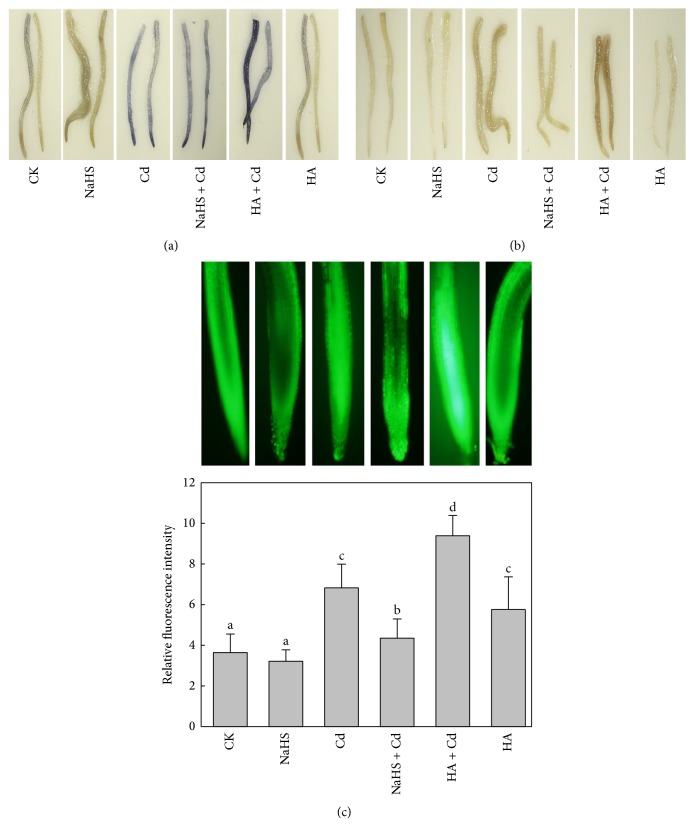
Effect of H_2_S on Cd-induced ROS accumulation in Chinese cabbage roots. (a) Histochemical detection of H_2_O_2_. (b) Histochemical detection of O_2_^•−^. (c) Fluorescence probe staining of ROS observed under fluorescence microscopy at 488 nm and relative fluorescence intensity of ROS. CK: control; NaHS: fumigated with 5 *μ*M NaHS for 24 h; Cd: 5 mM Cd stressed for 48 h; NaHS + Cd: seedlings fumigated with 5 *μ*M NaHS for 24 h and then treated with 5 mM Cd for 48 h; HA + Cd: seedlings treated with 1 mM HA for 4 h and then treated with 5 mM Cd for 48 h; HA: seedlings treated with 1 mM HA for 4 h. Data are mean ± SE of three independent repeats. LSD was used for multiple comparisons; different letters indicate significant differences (*P* < 0.05).

In order to determine whether H_2_S could regulate ROS content in roots to alleviate Cd-induced cell death, we colored H_2_O_2_ and O_2_^•−^ in the roots cells and measured ROS with a fluorescence probe. Notable accumulations of H_2_O_2_ and O_2_^•−^ took place in roots treated with 5 mM CdCl_2_ (Figures 4(a) and 4(b)). Both brown and blue precipitates were diminished in seedlings pretreated with NaHS for 24 h before 5 mM Cd treatment, compared with Cd treatment. Furthermore, the fluorescence intensity of ROS in roots treated with Cd was significantly increased by 62.8% (*P* < 0.05) compared with control. Simultaneously, NaHS + Cd treatment reduced ROS levels in roots by 36.9% (Figure 4(c)). The HA + Cd treatment even further increased the fluorescence intensity (by 34.5%) derived from Cd stress. Exogenous H_2_S significantly decreased the ROS accumulation caused by Cd stress. In contrast, pretreatment with an inhibitor of endogenous H_2_S increased the ROS content in roots.

### 3.5. H_2_S Reduced the Cd-Induced Accumulation of MDA and ELP

**Figure 5:**
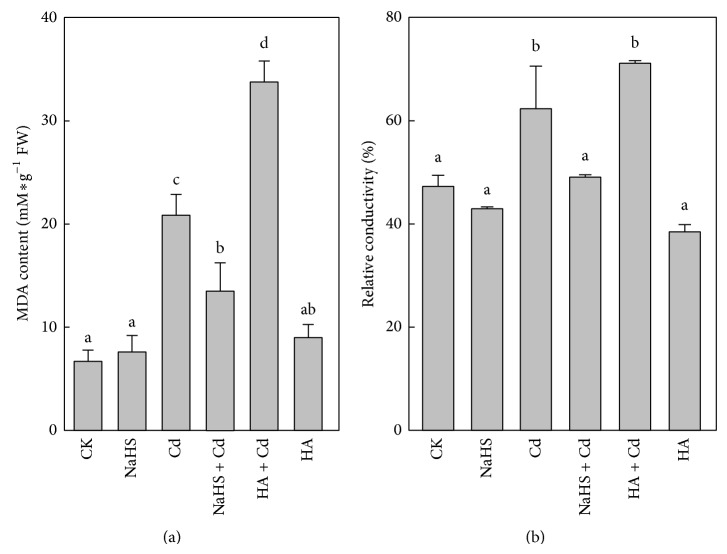
Effect of H_2_S on Cd-caused oxidation damage in Chinese cabbage roots. (a) Content of MDA. (b) Electrolyte leakage percentage. CK: control; NaHS: fumigated with 5 *μ*M NaHS for 24 h; Cd: 5 mM Cd stressed for 48 h; NaHS + Cd: seedlings fumigated with 5 *μ*M NaHS for 24 h and then treated with 5 mM Cd for 48 h; HA + Cd: seedlings treated with 1 mM HA for 4 h and then treated with 5 mM Cd for 48 h; HA: seedlings treated with 1 mM HA for 4 h. Data are mean ± SE of three independent repeats. LSD was used for multiple comparisons; different letters indicate significant differences (*P* < 0.05).

Exposure to 5 mM CdCl_2_ caused significant MDA overproduction, and NaHS + Cd significantly reduced MDA content (Figure 5(a)). Generation of MDA decreased by 35.4% in Chinese cabbage treated with NaHS + Cd compared with Cd treatment. In the presence of HA, MDA content following Cd treatment was about twice that for Cd alone.

There was a significant increase (42.5%) of ELP in roots for 48 h of Cd treatment compared with controls (Figure 5(b)). Roots with NaHS + Cd treatment showed a 27.7% decrease in ELP compared to Cd-exposed roots; and the HA + Cd treatment showed a slight increase compared with Cd treatment. As expected, 5 *μ*M NaHS pretreatment lowered the MDA content and the Cd-induced ELP. This result showed that suppression of cell membrane lipid peroxidation could guarantee structural integrity of the cells and improve plant tolerance to Cd stress.

### 3.6. H_2_S Regulated the Activity of Enzymes Scavenging ROS

The activities of SOD, CAT, POD, and APX were determined in an exploration of the effect of H_2_S on inhibition of ROS accumulation through enhancing activity of ROS-scavenging enzymes. There was a significant increase in activities of antioxidant enzymes (SOD, CAT, POD, and APX) in Chinese cabbage roots exposed to Cd. Activities of CAT in roots with NaHS + Cd treatment increased by 89% (*P* < 0.01) compared to Cd alone. SOD activity significantly increased 65% (*P* < 0.05) in NaHS + Cd treatment compared to Cd alone. Both SOD and CAT activities decreased with HA + Cd treatment. However, there was only slight increase being observed in APX and POD activities with NaHS + Cd treatment. Seedlings with NaHS treatment alone showed no significant effects on activity of the four antioxidant enzymes. Analysis of the four antioxidant enzymes revealed that the activities of CAT and SOD were more sensitive to regulating by H_2_S. The massive increase in activity of antioxidant enzymes could explain the significant decreases in ROS production.

## 4. Discussion

Numerous studies have focused on H_2_S as a regulator or a signal molecule in plants and its participation in response to diverse stresses. This study aimed to explore the mechanism of the positive effects of H_2_S on the plant growth inhibition caused by Cd. Cd stress has been reported to cause much physiological, biochemical, and structural damage in plants [16] with growth inhibition being the most directly perceived. We found that Cd-reduced growth inhibition was positively correlated with cell death in roots of Chinese cabbage (Figure 1). Cd treatment of tobacco cell cultures and onion roots eventually triggers either necrosis or PCD [18]. It is clear that Cd stress can induce cell death, but the mechanisms have remained unclear until now. However, the cells death mediated by ROS is notable [28]. In dead cells the orderly degradation of genomic DNA, which can be detected by DNA-ladder formation, is an important biochemical marker of PCD. In contrast, the random cleaving of nuclear DNA is a characteristic of cell necrosis [29]. Our results showed that genomic DNA was randomly degraded with 5 mM CdCl_2_ treatment, indicating induced acute cell necrosis in Chinese cabbage roots (Figure 3(b)).

In this experiment, we confirmed that Cd stress could result in the activation of cystein-desulfhydrase encoding genes transcription and H_2_S production (Figures 2(a) and 2(b)). Jin et al. [11] reported that drought stress triggered the expression of* AtLCD* and increase of the endogenous H_2_S production. In our present study, under Cd treatment, expression of* BraDES1* was mainly activated in roots of Chinese cabbage (Figures 2(a) and 2(b)). Both LCD and DES1 could specifically metabolize L-cysteine to form H_2_S, and pyridoxal phosphate was required as a cofactor. We speculate that H_2_S is generated through different pathways and helps plants responding to various stresses.

In Figure 2(c), seedlings were treated by different concentrations of NaHS and then Cd treatment. The data indicated that the elongation inhibition of Cd was significantly alleviated under 5 *μ*M NaHS pretreatment only. The pretreatment of 100 *μ*M NaHS had a toxicological effect on roots, and the difference was not observed in the other concentration of NaHS pretreatments. The results had consistency with the report that H_2_S has a narrowness of the transition zone between physiological and toxicological levels. As such, H_2_S can quickly cause the opposite effect when H_2_S concentration further increased [30].

Lipid peroxidation products are reportedly enhanced in shoot and root tissues of plants treated with Cd [31]. In this way, cell membranes may have their lipid composition modified and so their structure can be destroyed [32]. As mentioned above, H_2_S maybe protects the membrane integrity through depressing lipid peroxidation. Results provided evidence that H_2_S plays an important role in the response of Chinese cabbage to Cd stress. Seedlings pretreated with 5 *μ*M NaHS not only attenuated growth inhibition and cell death effectively but also decreased ROS accumulation significantly in roots upon 5 mM Cd treatment. Oxidative damage is represented by lipid peroxidation and ELP. Cd-induced ROS production was drastically decreased by NaHS treatment (Figure 4), consistent with significantly reducing MDA content and ELP in Cd-treated roots (Figure 5).

**Figure 6:**
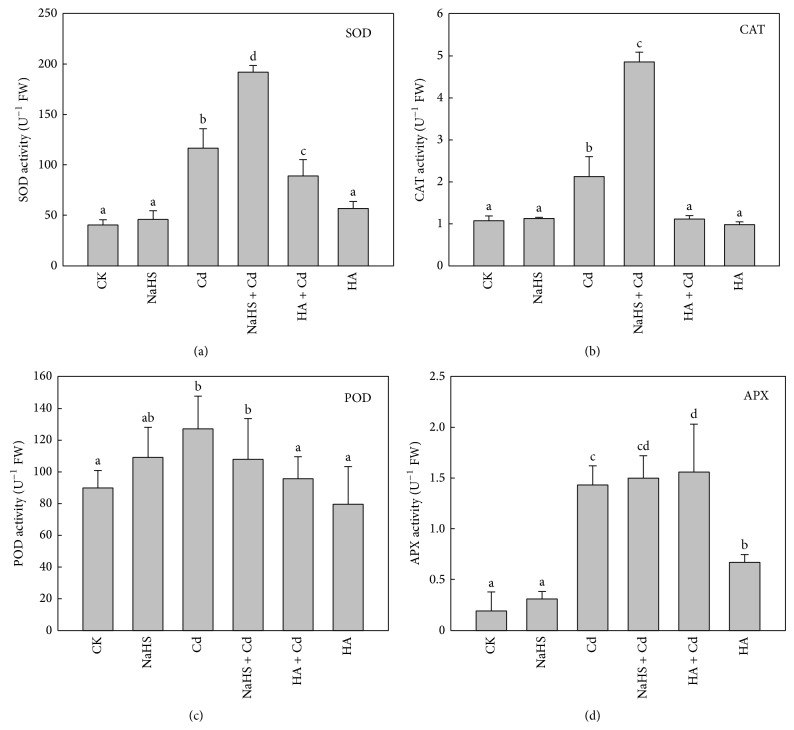
Effect of H_2_S on antioxidant enzyme activity in Chinese cabbage roots. Activities of (a) SOD, (b) CAT, (c) POD, and (d) APX. CK: control; NaHS: fumigated with 5 *μ*M NaHS for 24 h; Cd: 5 mM Cd stressed for 48 h; NaHS + Cd: seedlings fumigated with 5 *μ*M NaHS for 24 h and then treated with 5 mM Cd for 48 h; HA + Cd: seedlings treated with 1 mM HA for 4 h and then treated with 5 mM Cd for 48 h; HA: seedlings treated with 1 mM HA for 4 h. Data are mean ± SE of three independent repeats. LSD was used for multiple comparisons; different letters indicate significant differences (*P* < 0.05).

It was well known that, under Cd stress, ROS levels can rise to excessive levels, with oxidative damage and cell death as a consequence. Thus, continuous control of ROS and their metabolism is imperative under stress conditions. Plants have several antioxidant enzymes to scavenge ROS: SOD catalyzes superoxide radicals to H_2_O_2_ by very rapid dismutation; CAT and several kinds of peroxidases such as POD and APX then scavenge the H_2_O_2_ [33]. Chen reported that H_2_S significantly inhibited H_2_O_2_ and O_2_^•−^ production in leaves and roots of barely [13]. Our results demonstrated that H_2_S could significantly reduce the ROS accumulation caused by Cd stress (Figure 4). Current research has shown that, as a signal molecule, H_2_S regulates the metabolism and balance of ROS through upregulating the capacity of the antioxidant system to remove excess ROS. In this research, SOD, CAT, POD, and APX activities were determined to confirm the positive role of H_2_S in eliminating ROS. Our results showed that Cd stress could improve the activities of SOD, CAT, POD, and APX in roots. Meanwhile, NaHS + Cd treatment led to a significant increase in SOD and CAT activities. In contrast, POD and APX activities showed no difference between Cd and NaHS + Cd treatments. We concluded that H_2_S was involved in scavenging ROS accumulation via mainly enhancing SOD and CAT activities and partially stimulating POD and APX activities (Figures 6(a)–6(c)).

H_2_S-mediated tolerance to Cd stress in Chinese cabbage was related to the modulation of ROS homeostasis. However, H_2_S as a signal molecule and its embedded mechanisms of activating these antioxidant enzymes are little understood. In 2009, Mustafa et al. [34] performed the first study of protein S-sulfhydration in mammalian cells. Using a mass spectrometry assay they found that a large number of proteins were S-sulfhydrated under physiological conditions including glyceraldehyde-3-phosphate dehydrogenase, with its activity enhanced by protein S-sulfhydryl modulation. Catalase, related to elimination of H_2_O_2_, was also S-sulfhydrated. Since then, 176 proteins were identified in leaves of* A. thaliana* and some of them have also been found in mammalian systems [35]. Therefore, further study is needed to verify that sulfhydration of antioxidant enzymes is the signaling mechanism of H_2_S regulating these enzymes activities.

In conclusion, H_2_S had significant beneficial effects on Cd-exposed Chinese cabbage plants. It effectively blocked elevation of ROS, leading to activation of enzymes (SOD, CAT, POD, and APX) for ROS removal. H_2_S prevented plant from cell death caused by oxidant stress and so alleviated Cd-reduced growth inhibition.

## Supplementary Material

“The primers were used for real time-PCR. *LCD* (L-cysteine desulfhydrase), *DCD1* (D-cysteine desulfhydrase 1) and *DES1* (O-acetylserine(thiol)lyase homolog) were involved in H_2_S generation in Chinese cabbage. *ACTIN2* was used as an internal control.”
