# Supplementary material for: Hydrogen Sulfide Alleviates Cadmium-Induced Cell Death through Restraining ROS Accumulation in Roots of Brassica rapa L. ssp. pekinensis
Source: Oxid Med Cell Longev. 2015 May 11;2015:804603. doi: 10.1155/2015/804603 (PMC4442412; doi:10.1155/2015/804603)
Supplement: Supplementary file 1 — “The primers were used for real time-PCR. LCD (L-cysteine desulfhydrase), DCD1 (D-cysteine desulfhydrase 1) and DES1 (O-acetylserine(thiol)lyase homolog) were involved in H2S generation in Chinese cabbage. ACTIN2 was used as an internal control.” [file 804603.f1.pdf]

Supplemental materials

Table S1. List of all genes for qRT-PCR in the manuscript

Gene	Accession number	Primer pairs
ACTIN2	Bra022356	5' TCAGGTGTCCCGAGGTTCT 3'<br>5' TACTCATCCTATCAGCAATCCC 3'
LCD	Bra003488	5' AAGGTAGAGCGAACGGTAGAAC 3'<br>5' CTCACATAGTAATCAGCACCAA 3'
DCD1	Bra025184	5' AAGAGTTTACCAGGCAGTT3'<br>5' GCAAGACCAAACATCCC 3'
DES1	Bra036115	5' GGAGAACCAGGTCCACATT 3'<br>5' TTCCCACCAATAATCCTTC 3'
